# Supplementary material for: Alternative polyadenylation mediates genetic regulation of gene expression
Source: eLife. 2020 Jun 25;9:e57492. doi: 10.7554/eLife.57492 (PMC7338057; doi:10.7554/eLife.57492)
Supplement: Supplementary file 1. [file elife-57492-supp1.pdf]

Appendix:

3' Sequencing of nuclear mRNA captures mRNA species independent of mRNA decay:

To ensure that applying 3' Seq on the nuclear mRNA fraction would reflect polyadenylation usage of transcripts that have yet to be subject to decay, we verified that the nuclear mRNA 3' Seq captures features of nascent mRNA species prior to and independent from mRNA decay. To this end, we tested whether the ratio of nuclear to total mRNA 3' Seq reads correlates with measures of RNA decay. We reasoned that if nuclear mRNA captures mRNA species before they are subject to decay, then genes with more nuclear reads relative to total reads should have higher rates of mRNA decay. We used 4sU-seq (30m) data and RNA decay measurements collected in the same panel of lymphoblastoid cell lines (LCLs) as was used in this study as a proxy for mRNA rates of decay. The RNA decay and 4sU data were originally collected and processed in Pai et al. 2012 and Li et al. 2016, respectively. We further used RNA sequencing data collected in the same LCLs as used in this study and details regarding data processing can be found in Li et al. 2016.

We computed a score reflecting the nascent transcription rate for each gene as the normalized 4sU count over the sum of the RNA-seq and 4sU counts. This is because 4sU captures nascent mRNA that were metabolically labelled with a modified uridine. After a fixed amount of time (30min in this case), the modified transcripts are sequenced. A positive correlation between 4sU/RNA and nuclear/total 3' Seq across genes suggests that the nuclear 3' Seq captures polyadenylation usage at an earlier stage of the mRNA lifecycle.

In Li et al 2016, the authors presented a relationship between the same nascent transcription rate and a measure relative mRNA decay rate. They reported a negative correlation between nascent transcription and relative decay, whereby genes with faster nascent transcription also show faster rates of decay. We show a similar relationship between decay rate and our ratio of nuclear 3' Seq to nuclear and total mRNA 3' Seq, suggesting that we are capturing mRNA transcripts prior to mRNA decay in the nuclear fraction. To compute the correlations, we used the summary of the lm function in R.

Together, these correlations show that nuclear fraction 3'-Seq captures information that is not captured in 3' Seq from the total mRNA fraction, and importantly, that the difference is biologically rather than technically driven. Thus, we were able to use 3' Seq data from both nuclear and total mRNA fraction to study how genetic effects regulate APA at multiple stages of the mRNA lifecycle. In particular, the observed difference between APA in nuclear versus total mRNA fraction supports the notion that if genetic effects were detectable only in the total mRNA fraction, we should suspect that the genetic effect drives variation in post-transcriptional regulation such as decay or export. This assumption is based on the premise that mRNA from the total fraction better reflect mRNA diversity subsequent to decay and export. Because we do not see many examples of genetic effects only identified in the total mRNA fraction, we propose that nearly all genetic effect drive variation in APA co-transcriptionally.

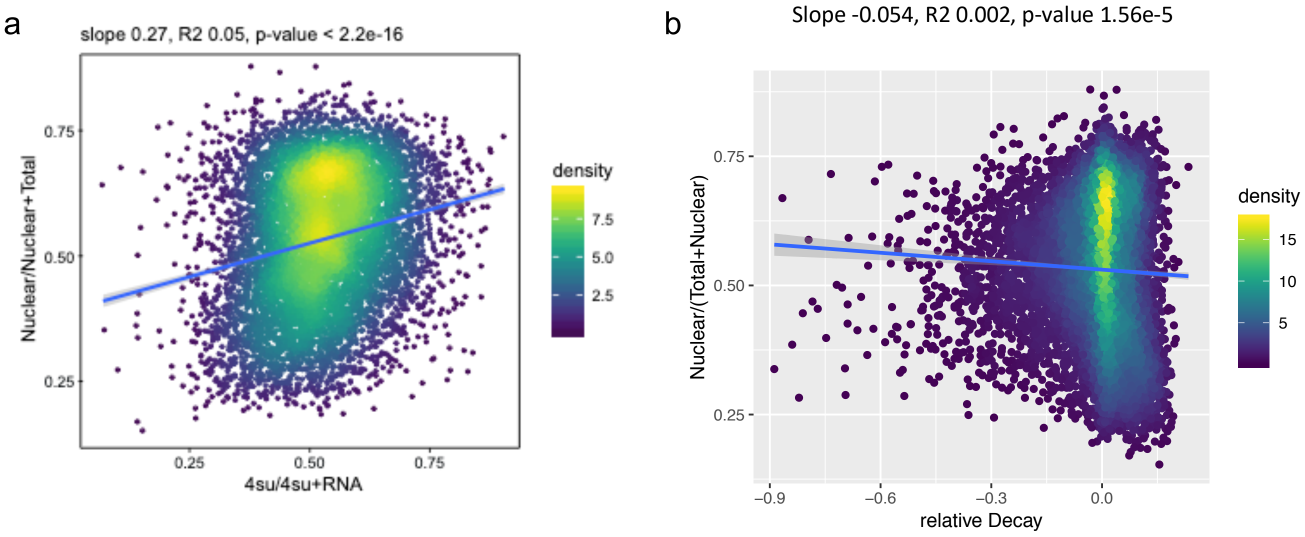

Appendix figure 1:

- **a.** Nuclear 3’ Seq captures polyadenylation of nascent transcripts. The ratio of new mRNA to steady-state mRNA (x axis) are plotted against the ratio of 3’ Seq reads from the nuclear fraction to 3’ Seq reads from the total mRNA fraction (y axis). Slope, R2, pvalue from a linear regression.
- **b.** Nuclear 3’ Seq captures polyadenylation of mRNA independent of mRNA decay. The relative decay rate of each gene (x axis) are plotted against the ratio of 3’ Seq reads from the nuclear fraction to 3’ Seq reads from the total mRNA fraction (y axis). Slope, R2, pvalue from a linear regression.

Intronic polyadenylation in other human tissues

In this study we used LCLs because of the rich molecular phenotyping that has been performed on the same cell lines. By collecting 3' Seq from cell nuclei we uncovered many more intronic PAS than expect. However, we are currently unable to validate whether these PAS are used in other human tissues because we are the first, to the best of our knowledge, to perform 3' Seq on mRNA from isolated nuclei in human cells.

That said, in order to estimate the extent to which intronic PAS we identified in the nuclear fraction are used in other human cell types, we turned to other APA studies that used a similar method to identify whole cell PAS. We reasoned that because total mRNA captures a small fraction of nuclear mRNA, it may be possible to use total mRNA to quantify the extent of intronic alternative polyadenylation in nuclei. For example, we found that 387 intronic PAS that were highly used in LCL nuclear mRNA were also detectable in LCL total mRNA. We can thus ask what fraction of these 387 intronic PAS also show evidence of usage in other cell-types from data collected by other studies on PAS. As baseline, we used 3' Seq usage data collected by Lianoglou et al., which include LCLs and four other cell-types (Breast, Ovary, Testes, Stem Cells). We found that about 10% of the 387 intronic PAS showed detectable usage in total 3' seq from LCLs collected by the Lianoglou study. By contrast, around 5% of the intronic PAS showed usage in Breast, and Testes. Usage of 3' Seq data from another study performed by Derti and colleagues suggest that nearly 10% of the 387 PAS showed detectable usage. Thus, these results suggest that there is at most a 2-fold difference in alternative polyadenylation in nuclei in other cell-types. While a 2-fold difference may appear large, we expect different cell-types to use different PAS depending on the specific genes that are expressed.

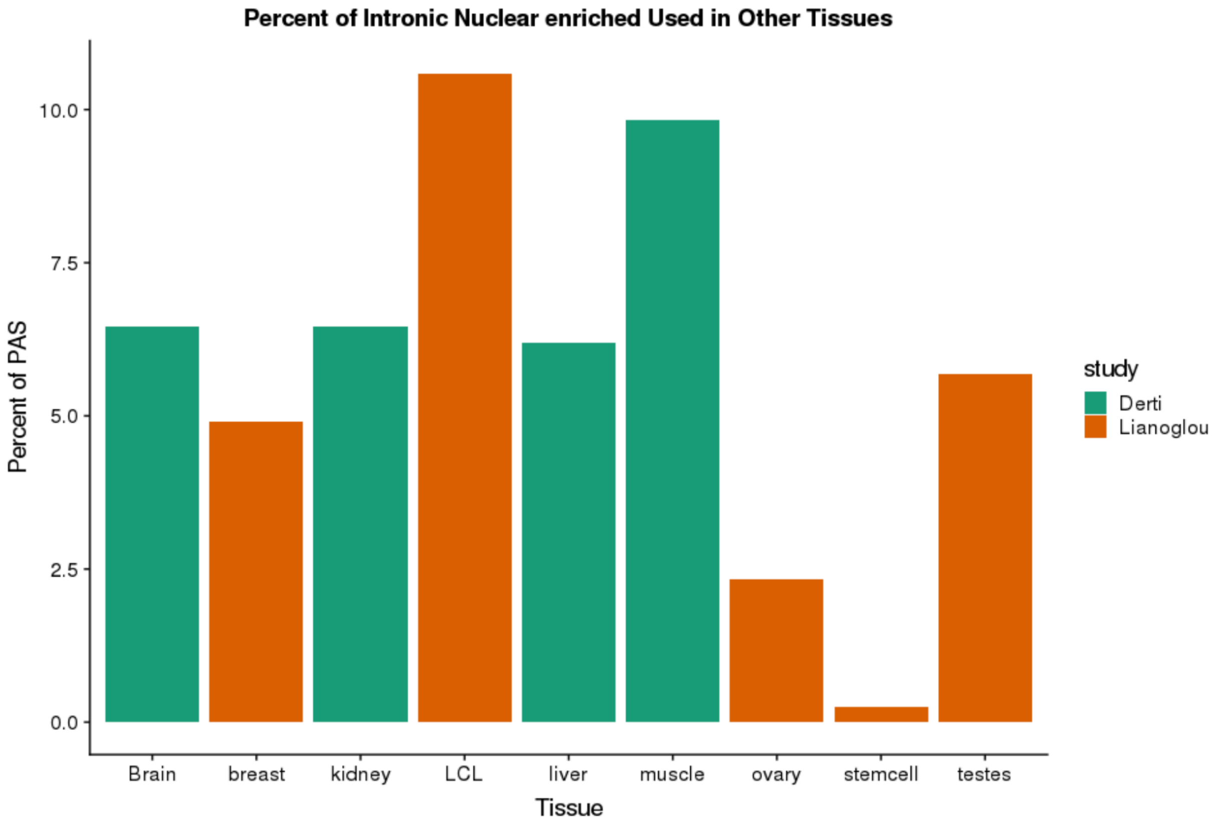

Appendix figure 2:

Intronic PAS enriched in the nuclear mRNA fraction of LCLs as detected in the total mRNA fraction of other human tissues. Barplot showing the percent of nuclear intronic PAS (of 387) discovered in whole cell 3' Seq from Derti et al., or Lianogou et al. Bar for each tissue is colored by study in which the data was collected.

RNA binding motifs:

3' UTRs are hotspots for RNA binding protein (RBP) motifs. When bound, RBPs can affect post transcriptional gene regulatory processes such as translation efficiency and nuclear export. We wanted to investigate whether genetic variants can impact APA by affecting binding of RBPs. To do this, we asked whether 3' UTRs with an apaQTL were more likely to be bound by an RBP than expected by chance. We downloaded eCLIP data for 25 RBPs collected by the ENCODE project in human K562 cells. We identified several RBPs enriched for genes with apaQTLs associated with 3' UTR PAS, but the overall enrichments were weak and are unlikely to explain the mechanism that underlie most apaQTLs. We did not see a similar enrichment for genes with intronic PAS apaQTLs. Interestingly, we found that the RNA binding proteins with the strongest enrichments are FUS and SAFB. These are intriguing result given the known function of FUS as a splice factor that guide nuclear export. We next asked if a genetic variant could be identified as an apaQTL due to differentially effects on one isoform but not the others. While we do not expect this to be the case genome wide, we do expect a small number of examples where a QTL could affect binding of an RBP and therefore isoform-specific post-transcriptional gene regulation. We identified 37 nuclear and 26 total apaQTLs overlapping eCLIP peaks. Of note, two apaQTLs disrupt binding for UPF1 which is a critical factor for nonsense mediated decay. A caveat to this analysis is the cell type specificity of RBP binding. eCLIP data is not available for LCLs.

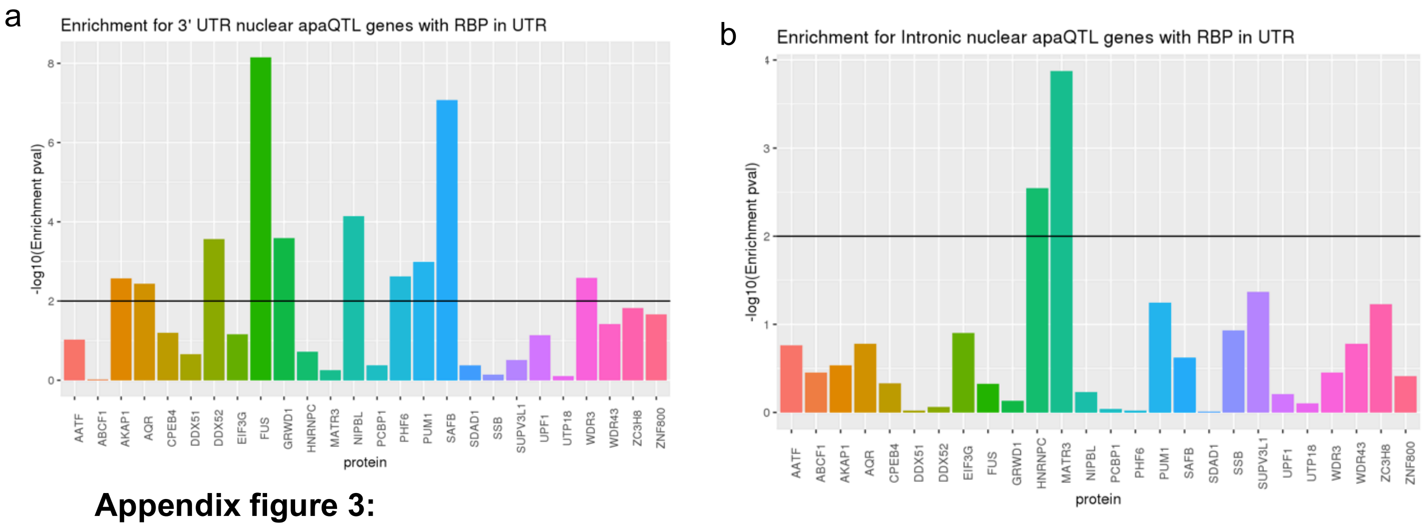

Appendix figure 3:

a. Enrichment for K562 cell RBP binding in 3' UTRs of genes with apaQTLs most strongly associated with a PAS in 3' UTRs compared to genes without apaQTL
b. Enrichment for K562 cell RBP binding in 3' UTRs of genes with apaQTLs most strongly associated with an intronic PAS compared to genes without apaQTL

Correlation between variance in ribosome occupancy and variance in APA

Variation in 3' UTR length can drive variation in translation efficiency. We wanted to test if this effect can be seen at the level of inter individual variation without requiring the existence of a QTL. We reasoned that if APA plays a role in modulating translation efficiency, then we would expect a correlation between APA variance and ribosome occupancy variance. When we correlated the variance in usage for the most highly used PAS for each gene, we see a weak but significant positive correlation between APA variance and ribosome occupancy variance (Correlation = 0.15, $p < 2.2\times10^{-16}$).

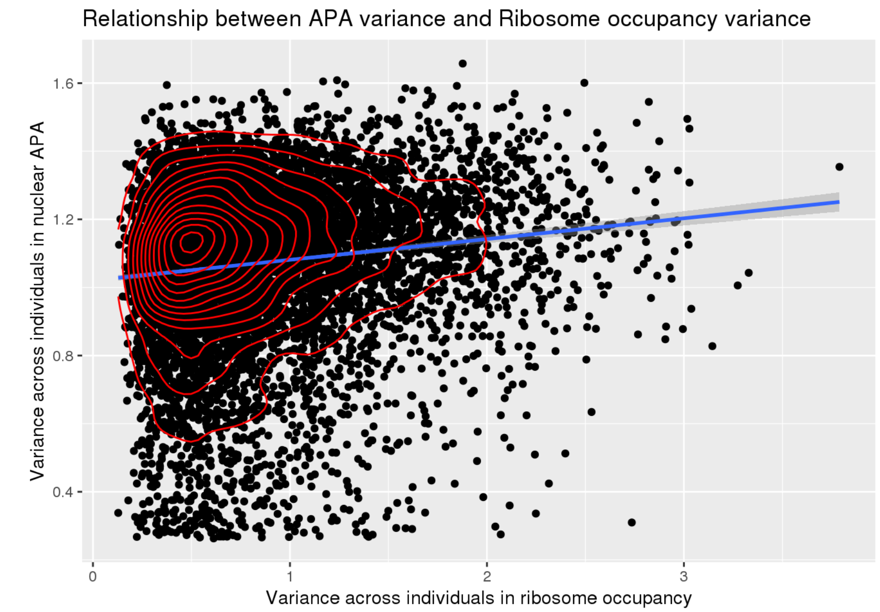

Appendix figure 4: Individual usage variance of the most highly used PAS in each gene (x axis) correlates with individual variance in ribosome occupancy (y axis) as measured in Li et al 2016.

Colocalization

In the main text we assert that APA can explain a proportion of the unexplained eQTLs, i.e. chromatin independent eQTLs. We primarily relied on correlation in order to draw this conclusion. However, to strengthen our claim, we used colocalization to ask if apaQTLs might generally be causal for the correlated eQTLs. To quantify the amount of colocalization between our apaQTLs and eQTLs, we used the COLOC package to test whether the apaQTL and eQTL associations share a causal SNP. The COLOC package estimates Bayes Factors for 4 alternative hypotheses. PP0: No association with either trait, PP1: No association with trait 1, PP2: No association with trait 2, PP3: Association with trait 1 and trait 2, two independent SNPs, and PP4: Association with trait 1 and trait 2, one shared SNP. If causal SNPs for an apaQTL and an eQTL is the same SNP, then PP4 is expected to be large > 0.5. One limitation of COLOC is that it is very sensitive to sample size and tend to assign large posterior probability to PP0, PP1, PP2 when either of the QTL mapping suffer from low power. This is because QTL mapping suffer from low power due to very small sample sizes compared to GWASs, for which coloc was designed for. To overcome this limitation, we used the ratio PP4/(PP3+PP4) to assess the colocalization probability instead of PP4/(PP0+PP1+PP2+PP3+PP4). To further increase power in our analysis, we used summary statistics from eQTLs identified on Geuvadis YRI LCL sample (n = 90) and used coloc to find colocalization between the eQTL signal and apaQTLs for the polyadenylation site (PAS) that is the most significant for the same gene. We expect this to be a lower bound for the actual number of colocalized eQTL-apaQTL SNPs because only one PAS for each gene is tested. Overall, we found that 33 genes had both and apaQTL and an eQTL and for which PP3+PP4 from coloc was 0.2 or greater. We found that the vast majority of genes (26, 78.8%) had a PP4/(PP3+PP4) value greater than 0.5, which indicates that the apaQTL and eQTL are more likely to share a causal SNP than not. Thus, we conclude that most apaQTLs that are determined to be eQTLs are likely to be causal, and further likely explain all the SNP effect on gene expression.

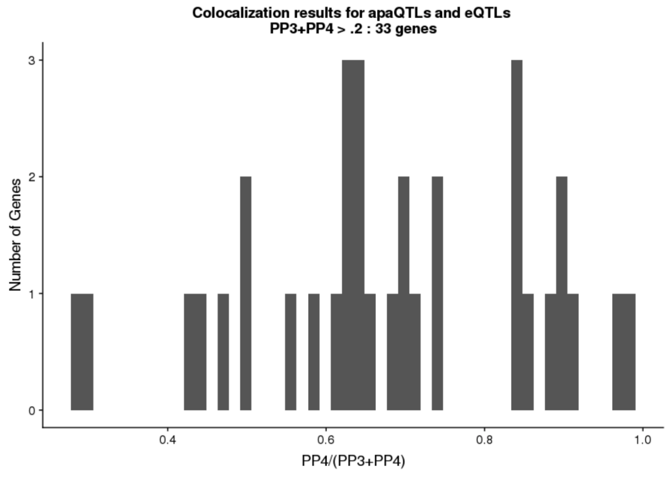

Appendix figure 5: The apaQTL and eQTLs for the large majority of genes that have both are more likely to colocalize than not. Histogram of number of genes with an apaQTL and eQTL for different values of PP4/(PP3 +PP4).

Evaluating the robustness of our finding to false positives caused by mispriming

We took various measures to ensure that misprimed reads are not included in our analysis. For example, we include filters both at the read and PAS level according to previous reports using the same experimental protocol (methods). In order to test if mispriming could still be responsible for the PAS we identified, we have looked at the base composition around our PAS. The results are below with 10 base pairs up and downstream of the PAS (PAS are at position 10 on plot). We have separated PAS based on their location and on whether the PAS is annotated in polyADB. We found a very similar base pair composition for all PAS except for intronic PAS that are unannotated in polyA DB. This suggests there may be some amount of mispriming for intronic PAS that are not annotated in the polyA DB. By quantifying the increase in A at nearby position around unannotated intronic PAS relative to annotated intronic PAS, we estimate that up to 20% of our unannotated intronic PAS may be explained by mispriming.

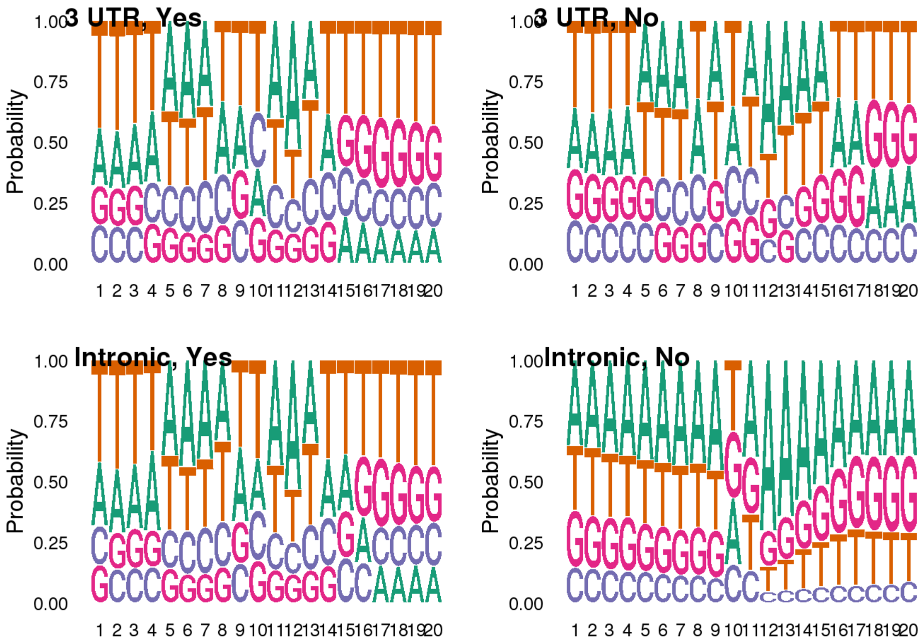

Appendix figure 6: Position weight matrices representing base composition 10 bps upstream and downstream of identified PAS separated by location and presence/absence of site in polyA DB.

However, we believe that the vast majority of unannotated intronic PAS are likely to be real. To support this view, we found that of the 9,605 unannotated intronic PAS, 24.6% have a canonical polyadenylation signal site upstream of the PAS. This matched the fraction of intronic PAS that are annotated, and is significantly higher than background (which is about 0.24%). Furthermore, the location of the canonical polyadenylation

signal site relative to the PAS location follows the expected distribution, which is 10-30bp upstream.

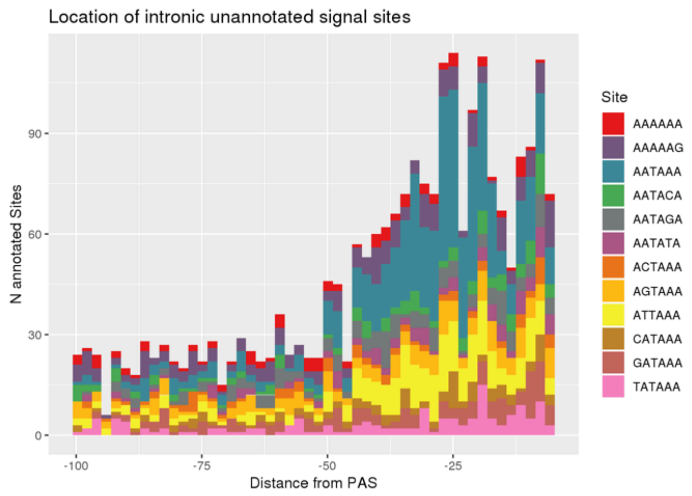

Appendix figure 7: Stacked histogram of polyadenylation signal sites upstream of unannotated intronic PAS. Distribution similar in shape and structure to that in Figure 1D.

While we would argue that a 20% rate of mispriming is reasonably low, and removing more PAS would lead to many false negatives, we nevertheless decided to rerun our analysis after removing intronic PAS that have not been previously annotated, to make sure that our results are robust to misprimed contaminates. We re-calculated the correlation between intronic effect sizes and eQTL effect sizes and found that the correlation is stronger than when the unannotated PAS are included (349 vs 357). This suggests that mispriming may be increasing noise.

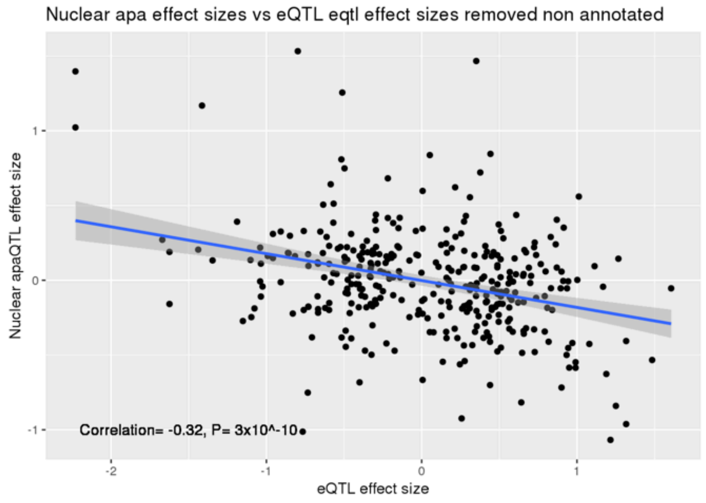

Appendix figure 8: Scatter plot of intronic apaQTL effect sizes after removing associations with unannotated intronic PAS plotted against their eQTL effect sizes. Supplemental to Figure 3A.

We also found that the proportion of eQTLs that are significant apaQTLs does not change dramatically (18% vs 17.3% of unexplained eQTLs using the 0.05 cutoff).

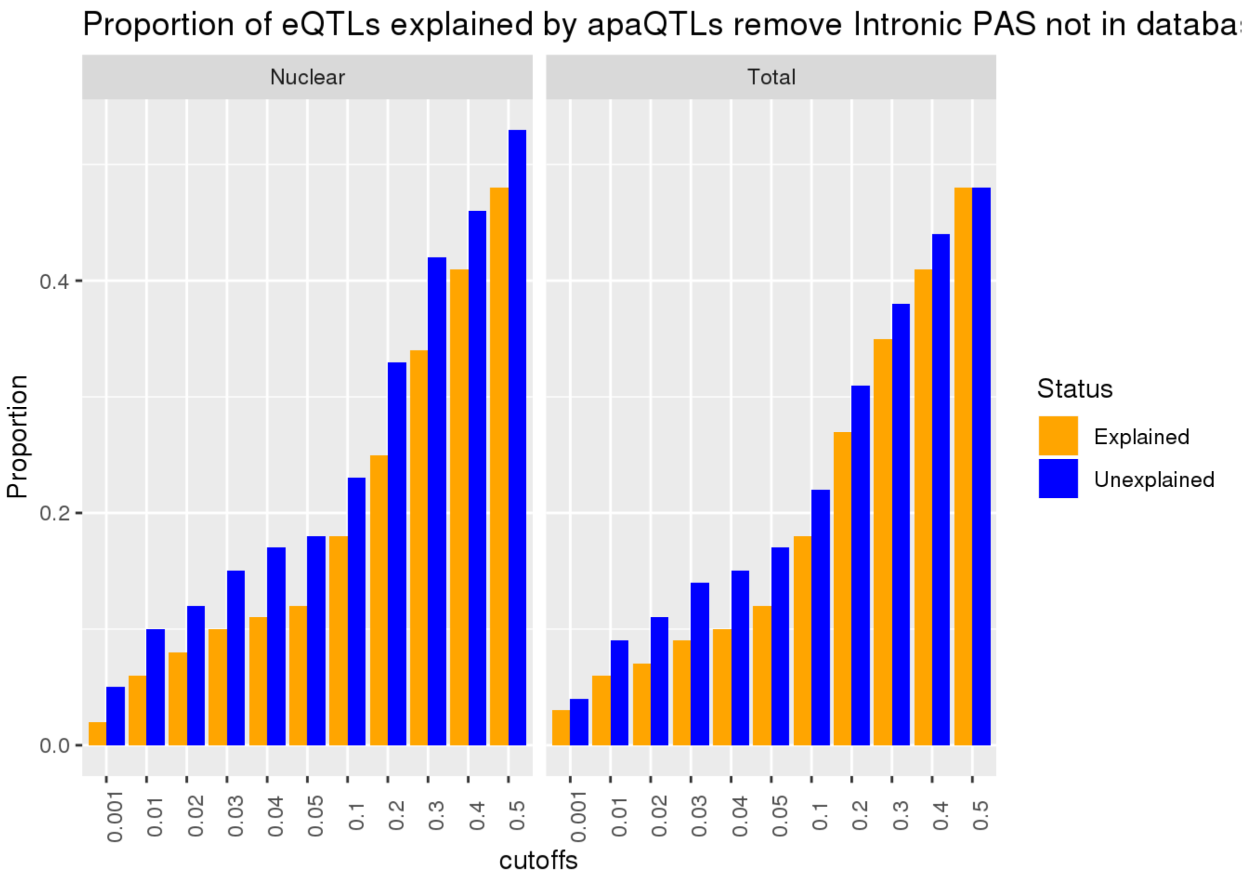

Appendix figure 9: Proportion of putatively explained by apaQTLs separated by fraction after removing associations with unannotated intronic PAS. Expression QTLs could be explained by apaQTLs identified from both fractions. This observation is robust to apaQTL association p-value cutoffs. We observed that apaQTLs explain a slightly higher proportion of previously unexplained eQTLs. Explained/Unexplained status of each eQTL was determined previously in Li et al. 2016.

Lastly, we found that nearly all apaQTLs that are not eQTLs but are associated with differences in translation and protein expression are not affected by the removal of unannotated intronic PAS (20 vs 25). Together these analyses suggest that even if our

set of intronic PAS include some false positives, these PAS do not drive the main conclusions of our work.
